# Advances in Nanomaterials Used in Co-Delivery of siRNA and Small Molecule Drugs for Cancer Treatment

**DOI:** 10.3390/nano11102467

**Published:** 2021-09-22

**Authors:** Shei Li Chung, Maxine Swee-Li Yee, Ling-Wei Hii, Wei-Meng Lim, Mui Yen Ho, Poi Sim Khiew, Chee-Onn Leong

**Affiliations:** 1 Nanotechnology Research Group, Faculty of Science and Engineering, University of Nottingham Malaysia Campus, Jalan Broga, Semenyih 43500, Selangor, Malaysia; edxsc1@nottingham.edu.my (S.L.C.); Poisim.Khiew@nottingham.edu.my (P.S.K.); 2 Department of Mechanical, Materials & Manufacturing Engineering, Faculty of Engineering, University of Nottingham Malaysia Campus, Jalan Broga, Semenyih 43500, Selangor, Malaysia; 3 Center for Cancer and Stem Cell Research, Institute for Research, Development and Innovation (IRDI), International Medical University, Kuala Lumpur 57000, Malaysia; lingweihii@imu.edu.my (L.-W.H.); weimeng_lim@imu.edu.my (W.-M.L.); 4 School of Pharmacy, International Medical University, Kuala Lumpur 57000, Malaysia; 5 Department of Materials Engineering, Faculty of Engineering and Technology, Tunku Abdul Rahman University College, Jalan Genting Kelang, Kuala Lumpur 53300, Malaysia; homy@tarc.edu.my; 6 Centre of Advanced Materials, Faculty of Engineering and Technology, Tunku Abdul Rahman University College, Jalan Genting Kelang, Kuala Lumpur 53300, Malaysia

**Keywords:** cancer treatment, drug-siRNA co-delivery systems, multifunctional nanocarrier, siRNA delivery

## Abstract

Recent advancements in nanotechnology have improved our understanding of cancer treatment and allowed the opportunity to develop novel delivery systems for cancer therapy. The biological complexities of cancer and tumour micro-environments have been shown to be highly challenging when treated with a single therapeutic approach. Current co-delivery systems which involve delivering small molecule drugs and short-interfering RNA (siRNA) have demonstrated the potential of effective suppression of tumour growth. It is worth noting that a considerable number of studies have demonstrated the synergistic effect of co-delivery systems combining siRNA and small molecule drugs, with promising results when compared to single-drug approaches. This review focuses on the recent advances in co-delivery of siRNA and small molecule drugs. The co-delivery systems are categorized based on the material classes of drug carriers. We discuss the critical properties of materials that enable co-delivery of two distinct anti-tumour agents with different properties. Key examples of co-delivery of drug/siRNA from the recent literature are highlighted and discussed. We summarize the current and emerging issues in this rapidly changing field of research in biomaterials for cancer treatments.

## 1. Introduction

Cancer is a large group of dreaded diseases in which abnormal cells proliferate at an uncontrolled rate. Metastasis is the invasion of malignant tumour cells to adjacent parts or other organs [1]. Globally, cancer is the major leading cause of premature deaths. In 2020 alone, 19.3 million new cancer cases were reported worldwide, with over 10 million deaths. A significant rise in cancer incidence of 47% is expected in 2040 [2]. Critically, provision of cancer care is one-half of a two-pronged global effort to reduce the overall burden of cancer [1,2].

Current cancer therapies include radiation, surgery, chemotherapy, targeted therapy or personalized medicine, as well as immunotherapy. Cancer is caused by mutations of genes that lead to activation of proto-oncogenes or inactivation of tumour suppressor genes [3]. Biomarker testing is a profiling of the tumour genetics, which enables treatments which target tumours with genetic changes in particular genes [4].

Chemotherapy drugs are commonly delivered directly through the oral, intravenous, or topical routes as well as through direct injections to the cancer site [4]. However, current chemotherapy targets only a limited subset of tumour-related molecules and pathways such as kinases, and DNA damage. Targeted therapy involves deploying small molecule drugs and monoclonal antibodies which targets the molecules which regulate tumour growth.

Recent advances in drug delivery systems have improved the efficacy of existing chemotherapy drugs by increasing their bioavailability to tumour sites. However, the fact remains that these drugs only target a few tumour-related factors but exclude tumour transcription factors. The majority of the signalling molecules in cancer is regulated by transcription factors (e.g., kRAS, p53, cJUN). Although drug molecules are unable to target these transcription factors, small- or short-interfering ribonucleic acid (siRNA) is able to interfere with their function.

siRNA has proven to be a useful tool to inhibit specific targets within tumour cells [5,6,7,8]. However, there are two significant issues with the use of siRNA alone for cancer treatment. Firstly, due to the specificity of siRNA, there is a high chance of tumour cells developing acquired resistance through further mutation. Secondly, as tumours are heterogenous in nature, even with the successful delivery of siRNA to a tumour, this alone does not guarantee the reduction of the tumour [8,9,10].

A viable solution for cancer treatment, therefore, consists of a combination of chemotherapy drugs and siRNA. A chemotherapy drug targets the bulk of a tumour (based on tumour-related phenotypes such as cell proliferation, DNA repair, etc.), whereas siRNA targets a specific mutation in a tumour. This justifies further development into co-delivery systems integrating small molecule chemotherapy drugs (molecular weight < 500 according to the National Cancer Institute) and siRNA.

Currently, various co-delivery systems are in various developmental stages. They consist of various classes of materials, including liposomes, dendrimers, as well as nanoparticles of polymeric, inorganic and metallic origins [11,12]. This review paper will summarize the desirable properties of these co-delivery systems, followed by a detailed report of different classes of co-delivery systems which host a combination of small molecule drugs and siRNA. A discussion of the advantages and disadvantages of each class of co-delivery system is presented as well. We also discuss current challenges faced in the systemic co-delivery of small molecule drugs-siRNA, and the strategies employed to overcome them. Finally, concluding remarks are provided to comment on the state of the art in this highly evolving and multidisciplinary field.

## 2. Desirable Properties of Co-Delivery Systems

A co-delivery system is a system which allows the concurrent delivery of more than one therapeutic substance. The therapeutic substances include chemotherapy drugs, deoxyribonucleic acids (DNAs), ribonucleic acids (RNAs), antibodies, etc.

Drug delivery systems enable the manipulation of drug properties, for instance, pharmaco-kinetics, pharmaco-dynamics, therapeutic index, biodistribution and cellular uptake of therapeutic agents. Various chemotherapeutics with poor water solubility, including Paclitaxel, require suitable delivery vehicles with high loading efficiency in order to successfully reach tumour cells [13]. 

In order to construct an effective and successful drug delivery system, several fundamental prerequisites are required: (i) the vehicles should be biocompatible, biodegradable, non-immunogenic, (ii) high loading capacity of and preservation of guest molecules, (iii) zero premature release before reaching and optimal uptake at the target site, (iv) effective endosomal escape, (v) controllable release rate, and (vi) active targeting of cells and tissues [5,14]. 

However, the requirements of a co-delivery system extend beyond the criteria listed above, as two agents with different physicochemical properties are incorporated in one drug carrier.

The vehicle for gene-delivery systems has additional criteria: (i) it must be capable of evading reticuloendothelial uptake [6,8], (ii) it must not engage in interaction with vascular endothelial cells and blood components, i.e., possess good stability or persistence in blood [5,6], and (iii) the system must be compact and stable enough to penetrate through the cell membrane and not degrade before reaching the nucleus [6,8,15]. The release mechanism from the vehicle is contingent on the types of oligonucleotides being transported. For instance, antisense oligodeoxyribonucleotides (ODNs) and siRNA should be targeted to be released in the cytosol to inhibit mRNA expression, while delivery of plasmid DNA should reach the nucleus as ectopic DNA [16]. In this review, the focus will be on the co-delivery of small molecule drugs and siRNA. 

As cancer belongs to the category of genetic diseases, siRNA-employed cancer treatment was reported to have significant results; however, the inherent characteristics of siRNA have elevated the difficulty of this type of therapy. Nucleic acid has an anionic hydrophilic structure with ~38 to 50 phosphate groups, which renders them impermeable through biological membranes [5,6,8]. Systemic delivered, unmodified naked siRNA is vulnerable to degradation through enzymatic digestion [17]. Besides that, siRNA has a molecular weight of ~13 kDa which is small enough for it to be easily removed through the kidney. However, its molecular weight is comparatively larger than small molecule drugs; this difference eventually affects the co-delivery system in terms of biodistribution and pharmacokinetics [7,8]. 

## 3. Classes of Co-Delivery Systems

Various combinations of small molecule drug-siRNA pairs have been reported in the literature to combat the highly heterogeneous and extremely complicated microtumour environment. There are strategies to formulate small molecule drug-siRNA combinations to improve the therapeutic efficacy of anticancer drugs: (i) to synergize the efficacy of anticancer drugs through synthetic lethality, (ii) to overcome multidrug resistance (MDR) silencing drug efflux pump-related gene, (iii) to enhance efficiency of cancer treatment by promoting apoptosis in cancer cells, and (iv) to promote anti-tumour therapy by targeting metastasis and angiogenesis [7].

**Figure 1:**
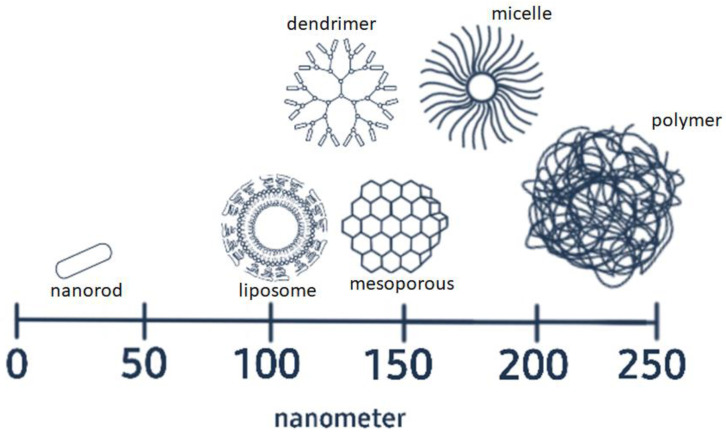
The structures and size range of vehicles used to co-deliver drug molecules and siRNA (not drawn to scale).

The emerging technology in nanomaterials development has introduced various options of drug carriers to be employed in co-delivery systems. Each class of materials has their own unique properties, conferring advantages as well as disadvantages for co-delivery. Figure 1 shows the structure and size range of various classes of materials commonly used in co-delivery applications. Each vehicle possesses distinctive structural features and morphology, which promote various advantages for co-delivery applications. Hollow or porous structures have better capability to encapsulate drugs, while surface modification of structures enables the surface attachment of drugs or siRNA. However, a common characteristic of all these structures is high surface-area-to-volume ratio.

The diversity and heterogeneity of cancer cells has made a tailored combination of chemotherapy drug-siRNA a highly promising treatment option. However, an appropriate vehicle is vital to a successful co-delivery system. Recent material choices for small molecule drug-siRNA co-delivery are reviewed in the following sections. A summary of the advantages and disadvantages of each co-delivery system is presented in Table 1.

### 3.1. Mesoporous Silica Nanoparticles

#### 3.1.1. General Properties

Mesoporous silica nanoparticles (MSNPs) are solid materials, in which hundreds of empty nanoscale channels are arranged in a two-dimensional network where the mesoporous structure is described as honeycomb-like [18,19]. By definition, the pores of mesoporous materials have a narrow pore size distribution ranging between 2–50 nm [20]. For instance, the MCM-41 mesoporous silica has a diameter of approximately 20 nm [21]. MSNPs have a manipulatable mesoporous structure, large pore volume, high specific surface area, and dual-functionality on the exterior and interior surfaces [19,22,23,24,25,26]. Their mesoporous inner structure provides for high drug loading capacity and enable control over drug release behaviour [19,25,26,27]. The unique properties of MSNPs enable the encapsulation of a wide range of therapeutic agents for targeted delivery, while preventing their premature release and degradation.

Silica is an abundant natural mineral having good biological compatibility. It is declared as “Generally Recognized As Safe” (GRAS) by the United States Food and Drug Authority (FDA). Silica materials are conventionally employed in industries such as cosmetics and food additives owing to their good biocompatibility [28]. Surface modification of the silica structure assists bioavailability and cellular uptake of the platform, avoids unwanted biological interactions, and bypasses monitoring by the immune system [22,27]. The theory of immune surveillance describes a patrol system to recognize and destroy invading pathogens as well as potential cancerous host cells [22,29,30].

Generally, the high surface-area-to-volume ratio and well-ordered mesoporous structure of MSNPs has been shown to facilitate improved drug loading capacity, biocompatibility and biodegradability [31,32,33]. The easily-functionalized surface properties of MSNPs allow their surfaces to be functionalized with siRNA to impart excellent colloidal stability [31,32]. These properties make MSNPs a promising vehicle for small molecule drug and siRNA co-delivery.

#### 3.1.2. Applications in Cancer Treatment

MSNPs offer the possibility of carrying drugs to the target site without degradation. However, it has been found that the drug released from the mesopores of unmodified MSNPs are often distributed off-target, an undesirable outcome for targeted drug delivery systems. Hence, the potential of MSNPs as co-delivery platforms have been expanded by the introduction of various surface modifications. The functionality of MSNPS has been enhanced from static drug vehicles into multifunctional delivery systems [18,19,25,26]. The ease of functionalizing the surfaces of MSNPs has resulted in MSNP-based systems which demonstrate properties such as sensitivity to pH, heat and enzymes, capability to undergo redox reactions, and responsiveness to magnetic fields. For instance, Yu et al. reported integrating superparamagnetic iron oxide nanocrystals in the core of MSNPs for magnetic hyperthermia cancer therapy applications [34].

**Figure 2:**
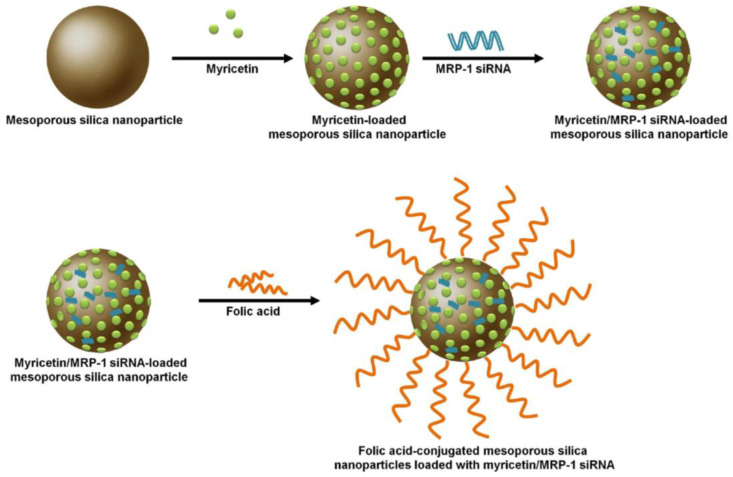
The preparation of folic acid-conjugated mesoporous silica nanoparticles loaded with myricetin and MRP-1 siRNA Note: MRP1 is equivalent to ATP binding cassette subfamily C member 1 (ABCC1). The MRP subfamily of genes is involved in multi-drug resistance. Reprinted from [31].

Song et al. [31] developed an MSNP vehicle loaded with myricetin (Myr) and MRP1-siRNA (Figure 2). The MSNP which was conjugated with folic acid (FA) showed increased specificity of Myr towards non-small cell lung cancer (NSCLC) compared with a non-targeted siRNA control. Sustained release of therapeutics was observed in the FA-conjugated vehicle compared with free Myr and the non-FA conjugated co-delivery system. Furthermore, the FA-conjugated co-delivery system caused increased apoptosis of lung cancer cells.

Wang and co-workers [10] prepared an MSNP co-delivery platform with doxorubicin and MDR1-siRNA and conducted in vivo studies on the impacts of the polyethylenimine (PEI)-coated MSNPs on oral cancer cells. They observed decreases in tumour size by 58.67 ± 2.37% when treated with the PEI-coated MSNPs loaded with doxorubicin alone and greater tumour reduction of 81.64 ± 3.17% when treated with the co-delivery system of drug and siRNA when compared to the control specimen. Their result suggested the effective inhibition of tumour growth in vivo when a co-delivery approach was applied.

Zhao et al. [32] found that an MSNP co-delivery system was able to improve tumour targeting efficiency of breast cancers. Their MSNP vehicle was modified with a disulfide group and loaded with doxorubicin and siRNA targeting BCL2 protein. It was reported that the doxorubicin content in tumour tissue after the co-delivery treatment was approximately 2.6-times greater than that of the free doxorubicin treatment. This indicated the potential of co-delivery systems in improving the enhanced permeability and retention (EPR) effect in breast tumour tissues. EPR describes a preferred accumulation of therapeutic agents in tumour cells [35,36].

However, Park et al. [36] stated that the heterogenous nature of tumours remain a challenge in targeted cancer therapy, even for nanoscale co-delivery platforms. Crucial factors that can enhance the EPR effect of cancer therapies include the application of molecular markers and enzymes that are specific to the tumour microenvironment (TME), as well as physical transformation of the TME.

Zhou et al. [37] reported a greater apoptotic rate (36.88%) when breast cancer cells were treated with an MSNP co-delivery system of doxorubicin and BCL2 siRNA, when compared with approximately 14% of apoptotic cells observed for the group treated with only doxorubicin loaded in the delivery system. The MSNP delivery system was modified with a copolymer of polyethylenimine-polylysine (PEI-PLL). The disulfide bonds on the copolymer were conjugated with a folate-linked poly(ethylene glycol) (FA-PEG). The resulting positively charged vehicle was able to bind with the negatively charged siRNA while encapsulating doxorubicin within the MSNP mesopores. Their results show that the co-delivery of doxorubicin and BCL2 siRNA offered synergistic cell apoptotic impacts on MDA-MB-231cells [37]. Table 2 shows various other examples of using MSNPs for co-delivery of small molecule drug and siRNA for the treatment of different types of cancer.

### 3.2. Polymeric Materials

#### 3.2.1. General Properties

Polymers are a large class of natural as well as synthetic materials, with a wide range of properties, which are directly related to their molecular weight, molecular structure and elemental composition. These macromolecules are made up of multiple repeating sub-units (mers). Polymer-based delivery systems are one of the most well-established platforms used to deliver therapeutic agents [38]. Their success is due to their versatility and tuneable characteristics in order to achieve the desired pharmaceutical and biomedical requirements [39].

Among the natural polymers which have been reported for use in drug delivery include derivatives of arginine, chitosan, cyclodextrin, polyglycolic acid (PGA), polylactic acid (PLA), and polysaccharides. These are generally favourable options due to their nontoxicity, biocompatibility and biodegradability [40,41]. PGA, PLA, polycaprolactone and polydioxanone are used as polymeric implant materials due to their biodegradable and bioabsorbable qualities [42].

On the other hand, man-made polymers such as poly(2-hydroxyethyl methacrylate (PHEMA) hydrogel, poly(n-isoproply acrylamide, polyethyleimine (PEI), and poly(n-(2-hydropropyl) methacrylamide) are preferred over the natural polymers as drug delivery systems due to their immunogenicity [42].

Amphiphilic block copolymers are a versatile class of self-assembling structures which can be formulated to produce polymeric nanoparticles such as. micelles and polymersomes [43]. Nanopolymeric delivery systems in micellar form alleviate the adverse effects of drugs, in which their hydrophobic core enables effective encapsulation of multiple anticancer agents including hydrophobic drugs [44,45]. The use of micelles was reported to lengthen drug retention time in the blood circulation and selectively accumulated in tumour tissue through the EPR effect [44,46].

Polymers bearing a positive charge or containing cationic functional groups in their structure are termed polycationic polymers. They have been extensively studied as a form of injectable, nonviral delivery agent for nucleic acid and drugs [40,47]. They also have wide application in biomedical areas, for instance as antimicrobial agents due to their affinity for the negatively-charged membrane of microorganisms, hence causing lysis of cell walls [48]. The most well-investigated synthetic linear cationic polymers include PEI, polyvinylpyrrolidone (PVP), and poly-L-lysine (PLL).

As a transfection agent, polycationic polymers are able to compress nucleic acid to form polyplexes for gene therapy. In general, to encapsulate and deliver a higher concentration of siRNA, polycations are engineered with greater charge densities, which are often associated with carrier-induced toxicity [49]. The toxicity of polymeric cation-mediated gene therapy is strongly linked to their chemical structure [50]. For instance, PEI with a low molecular weight (11.9 kDa) and a moderately branched structure showed about 100 times higher delivery efficiency, coupled with low toxicity, in comparison to a higher molecular weight PEI vector [51]. After delivery of the genetic payload, PEI is liberated of their cargo and has the potential to negatively interact with cellular components. Hence, an appropriate and suitable design of the polymeric drug carrier is vital. One solution to address delayed polymer toxicity issues is by incorporating PEG [50].

Copolymers of PLL-PEG are another example of thoughtful polymeric nanocarrier design. PLL are biodegradable, linear polypeptides in which the repeating unit is the amino acid lysine. PLL-nucleic acid polyplexes require the addition of chloroquine in order to improve their gene delivery efficiency. However, this results in increased cytotoxicity. With the use of higher molecular weight PLL, there is a trade-off between greater transfection efficiency and the accompanying higher cytotoxicity [52]. It has been shown that grafting of PEG to PLL can reduce cytotoxicity without reduction of transfection efficiency [53].

#### 3.2.2. Applications in Cancer Treatment

An emerging set of polymer-based drug delivery systems are being designed with moieties that are sensitive to changes in pH, temperature, the presence of glutathione, reactive oxygen species, and enzymes [54]. In the absence of disease, the bodily pH level is homeostatically maintained within a range of 7.35–7.45 [55]. Polymeric delivery systems can be designed to control the release of their therapeutic cargo by sensing the slightly acidic-pH levels linked to tumour microenvironments (pH 6.5–6.8) [44,46,56]. This particular property has gained popularity in the design of a controlled and targeted anticancer therapy system.

For instance, Suo et al. [56] applied a reversible addition-fragmentation chain transfer (RAFT) polymerization to obtain a triblock copolymer nanocarrier to mediate the delivery of doxorubicin and Bcl-2 siRNA to target breast cancer cells (MCF-7). Apart from offering a carrier for the delivery of the hydrophobic drug doxorubicin and siRNA, this synthetic method imparts excellent control of the nanocarrier physical properties, including uniformity in size, molecular weight, structure and reproducibility. The nanopolymeric carriers were able to achieve dual-release of its cargo in a reducing and acidic environment [56]. Their results demonstrated that a higher cellular uptake (51.6%) was noted in the cells treated with folate-modified co-delivery system, compared with the unmodified co-delivery system with only a 9.2% uptake. It was deduced that the targeted co-delivery promoted cellular uptake while anticancer effects were synergistically achieved by co-delivering two types of therapeutic agents.

**Figure 3:**
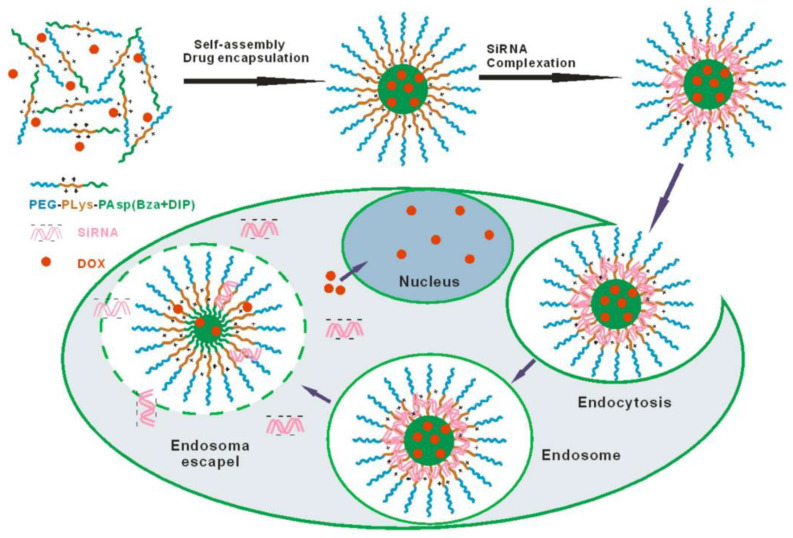
Illustration of self-assembly at pH 7.4 and intracellular releasing behaviour of amphiphilic block copolymer of monomethoxylpoly(ethylene glycol), poly(l-lysine), and poly(aspartyl(Benzylamine-co-(Diisopropylamino)ethylamine))mPEG-PLLys-PLAsp(BzA-co-DIP), abbreviated as PELABD micelles, for combinatorial delivery of hydrophobic anticancer drugs (Doxorubicin) and siRNA. Reprinted with permission from [46]. Copyright 2020 Springer Nature.

Li et al. [46] also reported a triblock copolymer delivery system in which the micelle size is controlled by changes in pH values; at acidic levels, the therapeutic agents are released in the tumour target. A schematic of the process of the self-assembly and therapeutics release of the co-delivery system designed by Li et al. [46] is shown in Figure 3. The triblock copolymer consisted of a PEG shell, a cationic PLL intermediate layer, and a pH-sensitive core of poly(aspartyl) (Benzylamine-co-(Diisopropylamino)ethylamine.

Additionally, Wu et al. [57] had constructed a type of co-delivering drug carrier with the use of triblock copolymer nanomicelles. The nanomicelles consisted of polyethylene glycol (PEG), polycaprolactone (PCL), and polyethylenimine (PEI). These core-shell nanomicelles were functionalized with folic acid (FA), then loaded with doxorubicin and P-glycoprotein (P-gp) siRNA to induce cell apoptosis in breast cancer cells. In vitro studies were carried out on MCF-7/ADR cell line. The flow cytometry results showed that the co-delivery system had increased the apoptosis level by 69.6% compared to the cells treated with free doxorubicin (85.3% vs. 15.7%) while 44% of increment in apoptosis level when compared to cells treated without the presence of P-gp siRNA (85.3% vs. 41.3%). These results confirm that this co-delivery system is able to deliver small molecule drug synchronously with siRNA. Furthermore, the release of siRNA was effective and able to interfere with the targeted expression [57].

In experiments conducted by Norouzi et al. [58], the best apoptosis induction was observed in dual functional polymeric micelles containing NF-κB (RELA) siRNA and gemcitabine (GemC18). Apoptosis was induced in 78% of MCF-7 cell lines and 95.4% in AsPC-1 cancer cells, illustrating co-delivery as the optimum delivery system for synergistic anticancer effects [58]. In another study, Yan et al. [44] developed a pH-responsive, chitosan-based polymer co-delivery system that delivered doxorubicin and Bcl-2 siRNA to actively target liver hepatocellular carcinoma cells, HepG2.

Table 3 shows a summary of the current combination of small molecule drug and siRNA co-delivery by using polymer-based nanomaterials for different types of cancer treatment.

### 3.3. Liposomes

#### 3.3.1. General Properties

Liposomes are soft spherical vesicles that are built mainly from phospholipid layers. They can be formed by one or more phospholipid layers where the layers are also called lamellae [68,69,70]. Phospholipids are under the class of lipids and can be found naturally in egg yolks (cholesterol) [70], vegetable oils, and can also be created synthetically [68]. Variations in phospholipids variations are mostly based on the modifications of head group or bondings formed; these are generally categorized according to the backbone groups–glycerol (glycerophospholipids) and sphingosine (sphingomyelins) [68]. These unique lipids are able to encapsulate a wide range of agents owing to their basic molecular structure which is composed of a hydrophilic head (polar head) and two hydrophilic tails (hydrocarbon chains) [68,70,71]. Liposomes are used as carriers for active agents with differing properties in a wide range of applications, such as cosmetics, pharmaceuticals, food, and farming. The wide-ranging applications of liposomes are attributed to their biocompatibility, encapsulation capability and modifiable formulation for the desired purpose [68,69,70,71,72].

The liposomal delivery system has attracted considerable attention in cancer therapy due to its unique properties to incorporate dissimilar therapeutic agents [59,73,74,75,76]. There are four main types of liposomes: (i) conventional liposomes, which includes cationic, anionic, phospholipids (neutral) and cholesterol, (ii) steric-stable liposomes, (iii) ligand targeting liposomes, and (iv) a combination of the first three types of liposomes [69]. The conventional liposomes are basic shields that are capable of protecting the payload (chemotherapy drugs), and also act as barriers to reduce the toxicity of the compounds in vivo [69]. The encapsulation property of liposomes is expected to mitigate the side effects of chemotherapy drugs. Besides that, its structure which resembles the plasma membrane of cells, plays a vital role in improving the cellular uptake of the compounds [68,72]. Furthermore, it was reported that with the use of liposome delivery systems, retention time in circulation is prolonged, alongside higher drug stability. Other benefits include a better controlled release profile when compared to the effect of using free chemical drugs alone [59,75,76].

However, the therapeutic efficacy of conventional liposomes is restricted by rapid clearance from the bloodstream in the reticuloendothelial system (RES), specifically through the liver and spleen [69]. The formulation can be modified with the use of hydrophilic polymers, such as polyethylene glycol (PEG), to obtain steric-stable liposomes, which are able to reduce in vivo opsonization and prolong circulation time in the bloodstream [69]. These liposomes are also reported to preferentially accumulate in the pathological sites and tumour regions by the EPR effect [69,74]

Liposomal delivery systems are not limited to drug delivery; they have been adopted for the incorporation of nucleic acid delivery, as well as in co-delivery of drugs and siRNA hybrid combinations. In using cationic liposomes (CLs), siRNA is protected from degradation by nuclease accumulation in the tumour, one of the key concerns in siRNA delivery. Anionic siRNA can be electrostatically bound to CLs to form a stable complex and improve the cellular uptake of target cells [69,77].

For advanced applications, site-specific or targeted-delivery can be achieved by attachment of ligands onto liposomes [69,70]. The ligand selections are based on the specific over-expression of ligands or specifically expressed ligands at the target cells, organs or tumours. Antibodies, peptides, proteins and carbohydrates are the general types of ligands that are commonly involved in the formulation of ligand-targeted liposomes [69]. Based on this fundamental concept, various types of liposomes have been developed. For instance, imaging agents can be attached onto liposomes to incorporate imaging properties. This conceptual theory has triggered the development of the “smart liposome”, in which the structure changes according to in vivo stimuli such as pH, temperature, enzyme, and magnetic field [77].

In general, all liposomes possess the fundamental properties required of delivery systems, such as, good biocompatibility, biodegradability, and low immunogenicity [59,76]. Liposome-based delivery systems also exhibit efficient loading of both hydrophobic drugs and genes, making them a promising candidate for co-delivery systems [76].

Liposomes were also reported as efficient in reducing cardiotoxicity of chemotherapy drugs such as doxorubicin, which damages heart muscle and function [75]. Besides that, liposomes are capable of penetrating the stratum corneum (outermost layer of skin) at different depths, compared to other nanocarriers [73,78]. The upper stratum corneum layer is the most penetrated layer while dioleoyl-phosphatidylethanolamine (DOPE)-based liposomes are able to achieve a deeper penetration [78]. Liposomes have been modified to improve their skin penetration ability for dermal drug delivery [79]. Deep penetration in the skin membrane is achievable via the use of transferosomes (liposomes with edge activators) or ethosomes (liposomes comprised of ethanol) [73]. These properties have made liposomes a great candidate for derma-related cancer treatment.

#### 3.3.2. Applications in Cancer Treatment

Jose et al. used a cationic liposome, 1,2-dioleoyl-3-trimethylammonium propane (DOTAP), for the co-delivery of curcumin and STAT3 siRNA to treat skin cancer [73]. The results showed the highest cell growth inhibition (76.3 ± 4.0%) in mouse melanoma cells (B16F10) compared to either curcumin-loaded liposomes or free STAT3 siRNA. This result suggested that this form of co-delivery was effective in curbing melanoma tumour progression.

Oh et al. studied galactosylated liposomes as a co-delivery vehicle to target hepatocellular carcinoma by loading it with doxorubicin and siRNA (Gal-DOX/siRNA-L). The Gal-DOX/siRNA-L system showed better doxorubicin uptake than free doxorubicin [75]. Besides that, the accumulated Gal-DOX/siRNA-L in tumour tissues was 4.8 times higher than that of free doxorubicin.

Lin et al. developed a co-delivery system from GE-11 peptide conjugated liposomes loaded with gemcitabine and siRNA as a formulation to target pancreatic cancer treatment [59]. They found that the co-delivery system displayed a 4-fold reduction in tumour weight compared to the control. When compared to GE-11 peptide conjugated liposome loaded with gemcitabine only (GE-GML), the tumour weight reduction was 2-fold.

Collectively, these results demonstrate the effectiveness of liposomal co-delivery system as an anticancer therapy. Table 4 shows the current combination of small molecule drug and siRNA co-delivery by using liposome nanomaterials for different types of cancer treatments.

### 3.4. Dendrimers

#### 3.4.1. General Properties

Dendrimers are a form of symmetrical, hyperbranched, low-molecular-weight polymers on the nanoscale, in which the architecture consists of a core, an inner shell, and an outer shell [80]. Its properties are dependent on functional groups which act as a capping agent on the outer shell [81]. The inner shell consists of several layers of repeating units (known as generations) built by a repetitive series of chemical reactions, while the outermost periphery contains multiple functional groups [82]. Polyamidoamine (PAMAM) dendrimers are one of the most well-studied dendrimers for delivery applications. Other types of dendrimers include peptide dendrimers (PPI), poly(l-lysine) dendrimers, and PAMAM-organosilicon dendrimers (PAMAMOS) [81].

Dendrimers are a versatile option as a vehicle for synergistic drug and gene combination therapy. Due to their unique and precise molecular structure, dendrimers can be used to deliver cancer drugs in any of the following ways: (1) cancer drugs can be covalently conjugated to the dendrimer outer shell to construct dendrimer prodrugs through direct coupling or cleavage linking, (2) encapsulating the drug within the central core to form a dendrimer-drug complex, and (3) exploiting the electrostatic interactions between functional groups on the dendrimer capping agent and the drug molecule [80,83].

Electrostatic interactions between the phosphate groups of siRNA and the cationic species on the dendrimer surface are crucial in the formation of complexes for effective delivery of gene therapy [84]. In comparison with conventional linear and branched polymers, dendrimers possess the following properties: (i) well-defined uniform spherical structure and manipulatable size in the nano-range, (ii) availability of numerous generations for different specific purposes [81,83], (iii) lipophilicity and suitable size enabling their diffusion through cell membranes [81], (iv) exceptional flexibility, and (v) low systemic toxicity [85,86]. Dendriplexes (dendrimers bound to nucleic acids) have an enhanced ability to evade endosomal entrapment due to the flexibility of dendrimers [81]. Endosomal escape is the process of siRNA exiting the endosome and entering the cytosol. This process is augmented by the “proton sponge” effect [81,85,87,88]. According to the “proton sponge” hypothesis, an influx of protons into the endosome results in increased buildup of osmotic pressure, causing destabilization of the endosomal membrane, leading to its rupture [88,89,90].

#### 3.4.2. Applications in Cancer Treatment

Table 5 shows the current combination of small molecule drug and siRNA co-delivery by using dendrimer-based nanomaterials for different types of cancer treatment.

Ghaffari et al. [91] studied the apoptotic effects of curcumin (Cur) and BCL2 siRNA co-delivered using polyamidoamine (PAMAM) dendrimers on HeLa cells. BCL2 siRNA was grafted to the amine groups on the surface layer of PAMAM while Cur was enveloped within the core to form the co-delivery dendriplex. Cells treated with PAMAM-Cur/siRNA showed an improvement of 58.77% as compared to cells treated with free curcumin alone. Compared with various formulations, the PAMAM-Cur/siRNA co-delivery system had the highest percentage of apoptotic cells among all formulations [91].

Li et al. [13] developed a hollow core/shell amphiphilic dendrimer engineered nanocarrier system (also known as ADENS) to co-deliver the hydrophobic drug paclitaxel and the hydrophilic siRNA. siRNA was loaded in the polar hydrophilic cavity while the paclitaxel was grafted in the polylactic acid (PLA) interlayer. The outer layer was constructed of polyethylene glycol (PEG) to enhance the in vivo circulation time of the co-delivery system and to avoid uptake by the reticuloendothelial system. ADENS was further modified with polypeptides which respond to tumour signals from the surrounding microenvironment. Results revealed that the system inhibited up to 73% of vascular endothelial growth factor (VEGF) at the mRNA level in A375 cell xenograft mice models when compared to controls; thus, demonstrating the synergistic effects of the co-delivery system.

### 3.5. Gold Nanoparticles

#### 3.5.1. General Properties

Nanoparticles of noble metal elements such as gold, silver and palladium have widespread use in biomedical applications [93]. Gold nanoparticles (AuNPs) in particular, have been extensively studied due to their versatile properties. Gold is one of the least reactive chemical elements and this has contributed to the properties of biocompatibility and biodegradability [94,95,96,97,98]. AuNPs possess tuneable optical properties, such as light scattering, localized surface plasmon resonance (SPR) and photothermal effects, leading to the incorporation of AuNPs in numerous medical diagnostics including ultrasensitive bioimaging and photothermal therapy [95]. The antimicrobial properties of AuNPs combined with their non-immunogenic and biocompatible properties have led to potential applications in cancer diagnosis and therapy as well as treatment for HIV infection [93]. Nanoparticles have at least one dimension measuring <100 nm and are defined by their high specific surface area. Gold has been synthesized in a variety of morphologies including nanorods, nanospheres, nanocages, nanoshells nanostars, nanorattles, nanopopcorns and nanoaggregates [95,96,99].

#### 3.5.2. Applications in Cancer Treatment

**Figure 4:**
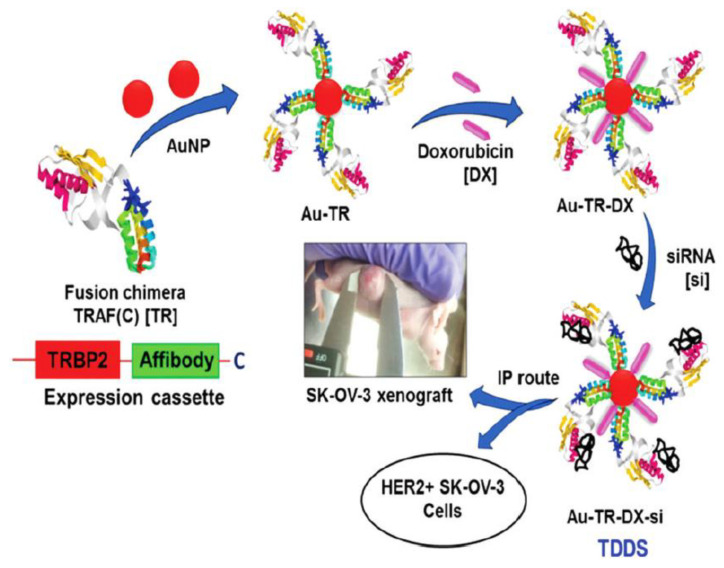
Fabrication of the AuNP-based targeted drug delivery system (TDDS) with an engineered bi-functional recombinant fusion protein TRAF(C) (TR), loaded with doxorubicin and ERBB2-siRNA (Au-TR-DX-si). It was used for further studies in vitro and *in vivo.* Reprinted with permission from [100]. Copyright Royal Society of Chemistry 2012.

The surface of AuNPs can be modified with active targeting ligands or moieties for tumour-specific targeting [95,96,98]. Through this strategy, passive accumulation and preferential retention of AuNPs at the tumour sites were observed, due to the EPR effect [94,98]. Recently, the distinctive properties of AuNPs have been explored in drug-gene co-delivery systems in cancer treatment. Kotcherlakota and co-workers reported when human ovarian (SK-OV-3) cells were treated with an engineered bi-functional recombinant fusion protein TRAF(C) (TR) gold nanoparticles (AuNPs), loaded with doxorubicin and erbB2-siRNA, a 6-fold difference in the tumour volume was observed as compared to the untreated control [100]. The AuNPs were conjugated with the reactive moiety at the carboxyl-terminus of the tumour necrosis factor (TNF) receptor associated factors (TRAF) protein. The authors described the synergistic effects of co-delivered doxorubicin and siRNA in inhibition of cell proliferation and tumour suppression. Figure 4 shows the fabrication of their co-delivery system based on AuNPs. Table 6 shows the current combination of small molecule drug and siRNA co-delivery by using gold nanoparticles nanomaterials for different types of cancer treatment.

## 4. Conclusions

There is an emerging trend in the use of combination therapy of drug-siRNA for cancer treatments. These experimental therapies rely increasingly on the use of nanostructured delivery vehicles to encapsulate and protect the therapeutics until it reaches the target. The most well-studied nanocarriers are mesoporous silica nanoparticles, dendrimers, polymers, liposomes and gold nanoparticles.

These structures fulfil the fundamental requirement of co-delivery systems of having low- or non-toxicity and biocompatibility. More complex challenges include tailoring their surface properties, designing suitable structures to entrap and protect the payload, prolonging their stability in the bloodstream, maximising targeted delivery and understanding their behaviour in the tumour environment. These are among the critical challenges and issues which need to be resolved before their successful implementation at the clinical level.

Given the advantage of co-delivering siRNA and drug simultaneously to achieve multi-target tumour therapy, there is a strong impetus for developing more materials that could improve the efficacy of these delivery systems. To conclude, the co-delivery of siRNA and small molecule drugs represent an emerging technology that warrants further investigation.

## Figures and Tables

**Table 1:** Summary of advantages and disadvantages of various systems used for co-delivery of small molecule drugs and siRNA.

Delivery System	Advantages	Disadvantages
Mesoporous Silica Nanoparticles	Naturally abundant Modifiable, nano-sized mesoporous structure Large pore volume High specific surface area Dual-functional exterior and interior surfaces Good biocompatibility and biodegradability Can be incorporated in magnetic applications Protects guest molecules before reaching target site	Extra modifications or coatings on surface for specific functions (targeted delivery, co-delivery of drugs and siRNA, sustained release of drug, etc.) Unsuitable for encapsulating drug molecules larger than the size of its pores Extra coating needed to enhance stability of drug in the carrier
Polymeric Materials	Cationic polymer encourages attachment of siRNA Hydrophobic core eases encapsulation Can be modified to have controlled release behaviours Can be designed to be sensitive to pH, temperature, chemical substances and enzymes Preferential accumulation in tumour tissues Various co-polymer combinations can be done to explore various potentials Protects guest molecules before reaching target site	Toxicity dependent on the chemical structure of polymers Appropriate design of polymer drug carrier is vital Modifications are needed for better performance Structure modification is required in certain conditions to reduce the cytotoxicity
Liposomes	Enables encapsulation of active agents Shielding effect reduces toxicity of chemotherapy drugs Improves cellular uptake Protects guest molecules before reaching target site Confers good drug stability Good controlled release profile Can be modified into cationic liposome for siRNA conjugation Manipulatable size Smart liposomes (sensitive to pH, temperature, enzymes and magnetic field) Imaging agents can be attached Good biocompatibility and biodegradability Low immunogenicity Able to penetrate the stratum corneum	Restricted by rapid clearance from the bloodstream in the reticuloendothelial system (RES) Modifications are needed to prolong circulation time in blood Ligand attachment is needed for site-specific delivery Appropriate selection is needed depending on the application
Dendrimers	Unique and precise molecular structure Conjugation of drugs through various types of bonding Uniform globular structure and molecular weight Wide range of generation number can be chosen for specific applications Manipulatable size and lipophilicity Can be engineered with ligands Low systemic toxicity Protects guest molecules before reaching target site	Properties are highly dependent on functional group of outer shell Performance of carrier is dependent on the appropriate functional group chosen Appropriate selection and method of encapsulation is crucial
Gold nanoparticles	Various morphologies available for different applications Variable optical properties Suitable for bioimaging applications Passive and preferential accumulation at tumour site High specific surface area Antimicrobial properties Good biocompatibility and biodegradability Least reactive element Ligands can be conjugated for various applications	High cost Rare Ligand attachment is needed for site-specific delivery

**Table 2:** Summary of co-delivery of small molecule drug and siRNA by mesoporous silica nanoparticles for cancer therapy.

Delivery System	Small Molecule Drug	siRNA Target	Type of Cancer	Cell Line	Testing Stage	Ref.
Mesoporous silica nanoparticles modified with polyethylenimine	Doxorubicin	ABCB1 (or MDR1)	Oral squamous carcinoma	KBV	*In vitro* *In vivo*	[10]
Folic acid (FA)-conjugated mesoporous silica nanoparticles	Myricetin	ABCC1 (or MRP1)	Non-small cell lung cancer	A594 NCI-H1299	*In vitro* *In vivo*	[31]
Mesoporous silica nanoparticles (MSNPs)	Doxorubicin	BCL2	Breast Cancer	MCF-7 HEK 293	*In vitro* *In vivo*	[32]
Acid-sensitive calcium phosphate/silica dioxide (CAP/SiO_2_) composite	Doxorubicin	ABCB1 (or MDR1)	Leukaemia	K562/ADR	*In vitro*	[33]
Mesoporous silica nanoparticles modified with polyethylenimine− polylysine and folate-linked poly(ethylene glycol)	Doxorubicin	BCL2	Breast Cancer	MDA-MB-231 RAW 264.7	*In vitro*	[37]

Note: ABCB1, ATP binding cassette subfamily B member 1; ABCC1, ATP binding cassette subfamily C member 1; BCL2, BCL2 apoptosis regulator; MDR1, multidrug resistance protein 1; MRP1, multidrug resistance associated protein 1.

**Table 3:** Summary of co-delivery of small molecule drug and siRNA mediated by polymer-based nanomaterials for cancer therapy.

Delivery System	Small Molecule Drug	siRNA Target	Type of Cancer	Cell Line	Testing Stage	Ref.
Chitosan based pH-responsive polymeric prodrug vector (GA-CS-PEI-HBA-DOX) *where GA-CS-PEI-HBA-DOX is prodrug chitosan-polyethylenimine-4-hydrazino-benzoic acid doxorubicin*	Doxorubicin	BCL2	Liver cancer	HUVEC, HepG2	*In vitro* *In vivo*	[44]
Amphiphilic block copolymer of monomethoxylpoly(ethylene glycol), poly(l-lysine), and poly(aspartyl(Benzylamine-co-(Diisopropylamino)ethylamine)) mPEG-PLLys-PLAsp(BzA-co-DIP), abbreviated as PELABD micelles	Doxorubicin	siRNA	Ovarian cancer	SKOV3	*In vitro*	[46]
Triblock copolymer nanocarrier of PAH-b-PDMAPMA-b-PAH *where PAH-b-PDMAPMA-b-PAH is poly(acrylhydrazine)-block-poly(3-dimethylaminopropyl* *methacrylamide)-block-poly(acrylhydrazine) (PAH-b-PDMAPMA-b-PAH)*	Doxorubicin	BCL2	Breast cancer	MCF-7	*In vitro*	[56]
PEG-PCL-PEI triblock copolymer nanomicelle functionalized with folic acid	Doxorubicin	(P-gp) siRNA	Breast cancer	MCF-7/ADR	*In vitro*	[57]
Poly(ε-caprolactone), polyethylenimine and polyethylene glycol (PCL-PEI-PEG) copolymers	4-(N)-stearoyl gemcitabine	RELA	Pancreatic cancer and breast cancer	AsPC-1, MCF-7	*In vitro*	[58]
Lipid-polymer hybrid nanoparticles *where cationic ε-polylysine co-polymer nanoparticles (ENPs) are coated with PEGylated lipid bilayer resulted formation of LENPs, with reversed surface charge*	Gemcitabine	HIF1A	Pancreatic cancer	Panc-1	*In vitro* *In vivo*	[59]
pH/redox dual-sensitive polymeric materials (cPCPL) *where cPCPL is poly(ethylene glycol))x-(chitosan-polymine)y-(lipoic acid)z grafted with cRGDyC-PEG-NHS, cRGDyC is a kind of peptide, PEG is poly(ethylene glycol) and NHS is hydroxysuccinimide.*	Etoposide	EZH2	Orthotopic non-small-cell lung tumour	luc-A549	*In vitro* *In vivo*	[60]
Self-assembled polyjuglanin nanoparticles (PJAD-PEG) *where PJAD-PEG is poly(juglanin (Jug) dithiodipropionic acid (DA))-b-poly(ethylene glycol) (PEG)*	Doxorubicin	KRAS	Lung cancer	A549, H69	*In vitro* *In vivo*	[61]
Cationic polyethylenimine-block-polylactic acid (PEI-PLA)	Paclitaxel	BIRC5	Lung Adenocarcinoma	4T1, A549	*In vitro* *In vivo*	[62]
Lactic-co-glycolic acid (PLGA) nanoparticles	Paclitaxel	SPDYE7P	Cervical cancer	HeLa	*In vitro* *In vivo*	[63]
FeCo-polyethylenimine (FeCo-PEI) nanoparticles and polylactic acid-polyethylene glycol (PLA-PEG)	Paclitaxel	FAM group	Breast cancer	MCF-7, BT-474	*In vitro*	[64]
Hypoxia-sensitive PEG-azobenzene-PEI-DOPE (PAPD) nanoparticles	Doxorubicin	ABCB1	Ovarian cancer and breast cancer	A2780 ADR, MCF7 ADR	*In vitro*	[65]
Chondroitin sulfate (CS)-coated β-cyclodextrin polyethylenemine polymer	Paclitaxel	MCAM	Breast Cancer	MDA-MB-231, MCF-7	*In vitro*	[66]
Targeted multifunctional polymeric micelle (TMPM) *where TMPM is made up of triblock copolymer poly(ε-caprolactone)-polyethyleneglycol-poly(L-histidine) (PCL-PEGPHIS)*	Paclitaxel	VEGF group	Breast Cancer	HUVECs, MCF-7	*In vitro*	[67]

Note: BCL2, BCL2 apoptosis regulator; HIF1A, hypoxia inducible factor 1 subunit alpha; EZH2, enhancer of zeste 2 polycomb repressive complex 2 subunit; KRAS, KRAS proto-oncogene, GTPase; BIRC5, baculoviral IAP repeat containing 5; SPDYE7P, speedy/RINGO cell cycle regulator family member E7 pseudogene; FAM group, long non-coding family with sequence group; ABCB1, ATP binding cassette subfamily B member 1; MCAM, melanoma cell adhesion molecule; VEGF group, vascular endothelial growth factor group; RELA, RELA proto-oncogene, NF-kB subunit.

**Table 4:** Summary of co-delivery of small molecule drug and siRNA by liposomes for cancer therapy.

Delivery System	Small Molecule Drug	siRNA Target	Types of Cancer	Cell Line	Testing Stage	Ref.
GE-11 peptide conjugated liposome	Gemcitabine	HIF1A	Pancreatic cancer	Panc-1	*In vitro* *In vivo*	[59]
1,2-Dioleoyl-3-trimethylammonium propane (DOTAP) -based cationic liposomes	Curcumin	STAT3	Skin cancer	B16F10	*In vitro* *In vivo*	[73]
PEGylated liposomes	Docetaxel	BCL2	Lung cancer	A549, H226	*In vitro* *In vivo*	[74]
Galactosylated Liposomes	Doxorubicin	VIM	Hepatocellular Carcinoma	Huh7, A549	*In vitro* *In vivo*	[75]
Carbamate-linked cationic lipid (Cationic Liposome)	Paclitaxel	BIRC5	Lung Cancer	NCI-H460	*In vitro*	[76]

Note: STAT3, signal transducer and activator of transcription 3; BCL2, BCL2 apoptosis regulator; VIM, vimentin; HIF1A, hypoxia inducible factor 1 subunit alpha; BIRC5, baculoviral IAP repeat containing 5.

**Table 5:** Summary of co-delivery of small molecule drug and siRNA by dendrimer-based nanomaterials for cancer therapy.

Delivery System	Small Molecule Drug	siRNA Target	Type of Cancer	Cell Line	Testing Stage	Ref.
Amphiphilic dendrimer engineered nanocarrier system (ADENS) modified by tumour microenvironment-sensitive polypeptides (TMSP) (TMSP-ADENS)	Paclitaxel	FAM and VEGF group	Melanoma, prostate cancer	A375, PC-3, HT-1080	*In vitro* *In vivo*	[13]
PTP (plectin-1 targeted peptide, NH2-KTLLPTP-COOH), biomarker for pancreatic cancer, integrated with the PSPG vector to form peptide-conjugated PSPG (PSPGP) *where PSPG is branched poly(ethylene glycol) with G2 dendrimers through disulfide linkages*	Paclitaxel	NR4A1 (or TR3)	Pancreatic Cancer	Panc-1	*In vitro* *In vivo*	[83]
Hyaluronic acid (HA) modified MDMs *where MDMs is the PAMAM-PEG2k-DOPE co-polymer, together with mPEG2k-DOPE, was formulated into mixed dendrimer micelles, and PAMAM is the generation 4 polyamidoamine*	Doxorubicin	ABCB1 (or MDR1)	Ovarian cancer, colorectal carcinoma and breast cancer	A2780 ADR, HCT 116, MDA-MB-231	*In vitro*	[86]
PAMAM-OH derivative (PAMSPF)	Murine double minute 2 protein (MDM2) inhibitor RG7388	TP53	Breast cancer	P53-wild type MCF-7 cells (MCF-7/WT), MDA-MB-435	*In vitro* *In vivo*	[87]
Polyamidoamine (PAMAM) dendrimer	Curcumin	BCL2	Cervical cancer	HeLa	*In vitro*	[91]
Folate-polyethylene glycol appended dendrimer conjugate with glucuronylglucosyl-β cyclodextrin (Fol-PEG-GUG-β-CDE) (generation 3)	Doxorubicin	PLK1	Cervical cancer	KB	*In vitro* *In vivo*	[92]

Note: BCL2, BCL2 apoptosis regulator; NR4A1, nuclear receptor subfamily 4 group A member 1; TR3, thioredoxin reductase 3; ABCB1, ATP binding cassette subfamily B member 1; MDR1, multidrug resistance protein 1; FAM, long non-coding family with sequence group; VEGF, vascular endothelial growth factor group; TP53, tumour protein p53.

**Table 6:** Summary of co-delivery of small molecule drug and siRNA by gold nanomaterials for cancer therapy.

Delivery System	Small Molecule Drug	siRNA Target	Type of Cancer	Cell Line	Testing Stage	Ref.
Polyelectrolyte polymers coated gold nanorods (AuNRs)	Doxorubicin	KRAS	Pancreatic Cancer	Panc-1	*In vitro* *In vivo*	[95]
Gold nanoparticles (AuNPs) combined with an engineered bi-functional recombinant fusion protein TRAF(C) (TR)	Doxorubicin	ERBB2	Ovarian cancer	SK-OV-3, MDA-MB-231, A549, PANC-1, B16F10	*In vitro* *In vivo*	[100]
Layer-by-layer assembled gold nanoparticles (LbL-AuNP)	Imatinib mesylate	STAT3	Melanoma cancer	B16F10	*In vivo*	[101]

Note: KRAS, KRAS proto-oncogene, GTPase; ERBB2, erb-b2 receptor tyrosine kinase 2; STAT3, signal transducer and activator of transcription 3.
